# Supplementary material for: A Green Bioactive By-Product Almond Skin Functional Extract for Developing Nutraceutical Formulations with Potential Antimetabolic Activity
Source: Molecules. 2023 Dec 3;28(23):7913. doi: 10.3390/molecules28237913 (PMC10708410; doi:10.3390/molecules28237913)
Supplement: Supplementary file 1 [file molecules-28-07913-s001.zip › molecules-2693027-supplementary.pdf]

Supplementary material

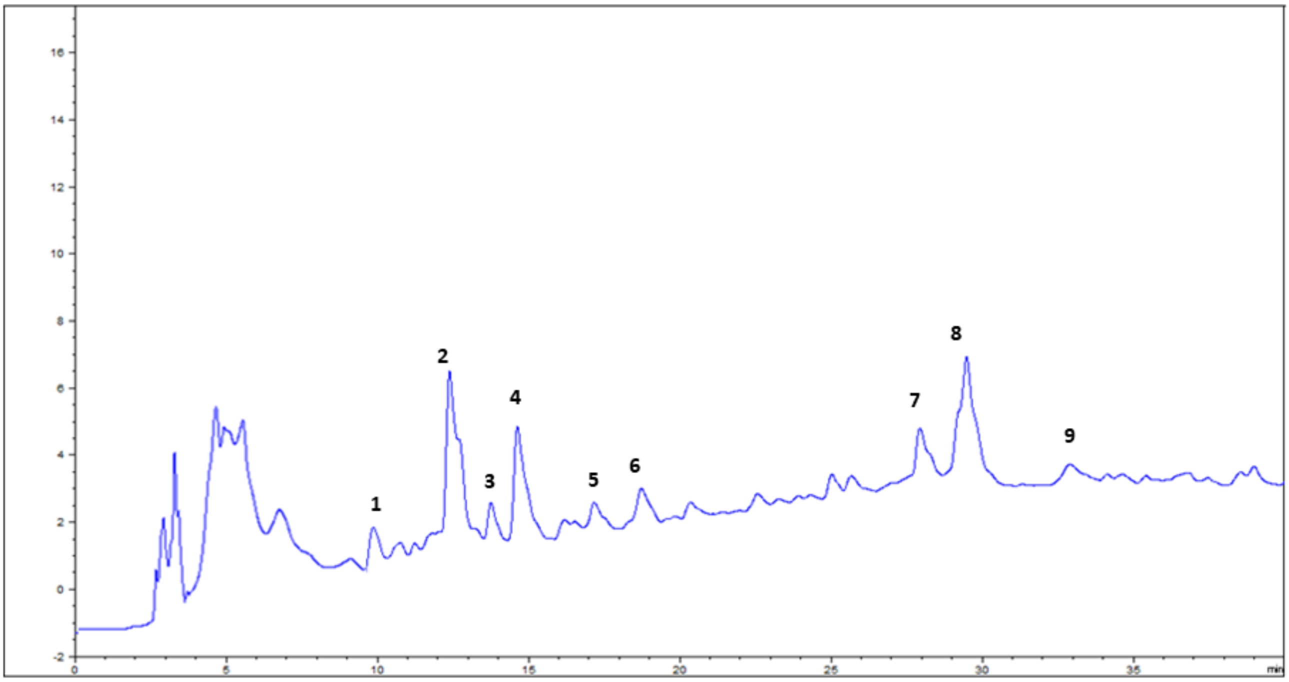

Figure S1. HPLC-UV profile of DSA lio: Gallic acid (**1**) procyanidin B3 (**2**), procyanidin B1 (**3**), catechin (**4**), epicatechin (**5**), procyanidin C1 (**6**), kaempferol 3-*O*-rutinoside (7), isorhamnetin-3-*O*-rutinoside (**8**), and quercetin (**9**).

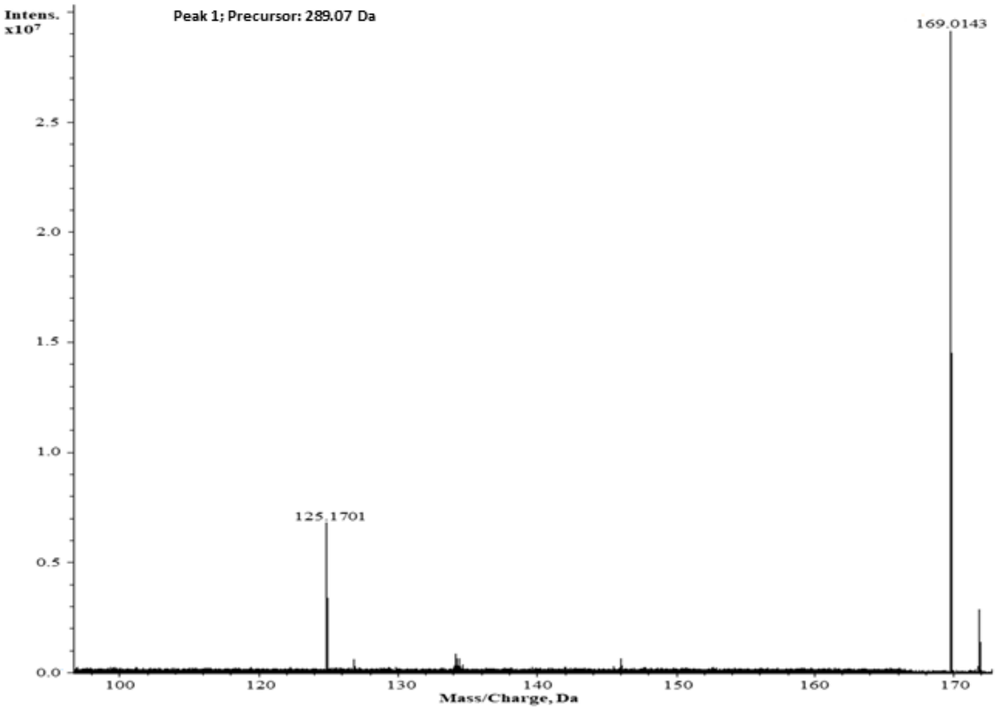

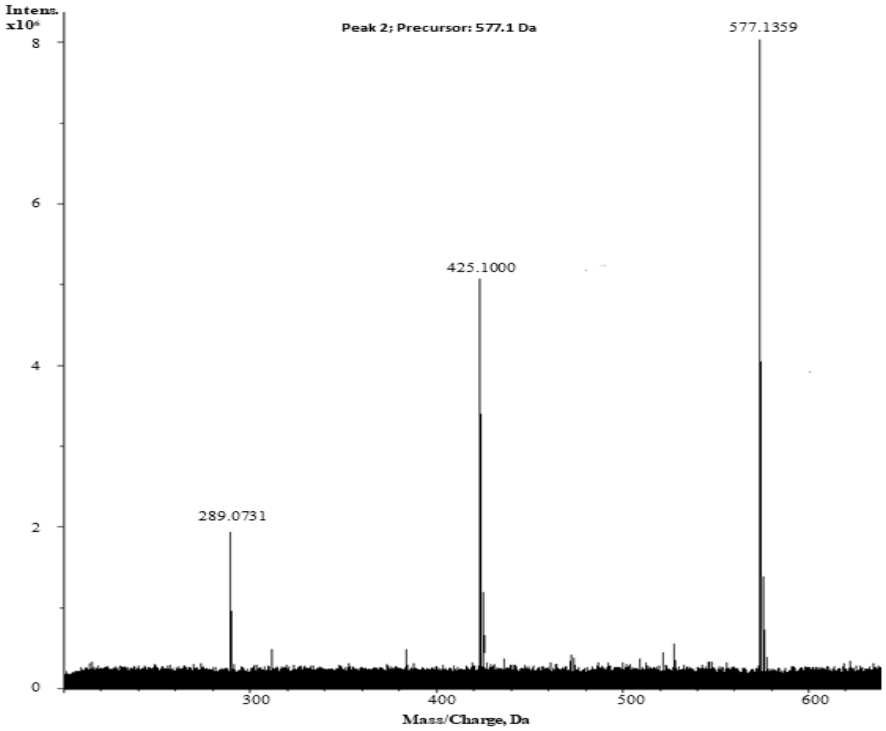

Figure S2 (continued)

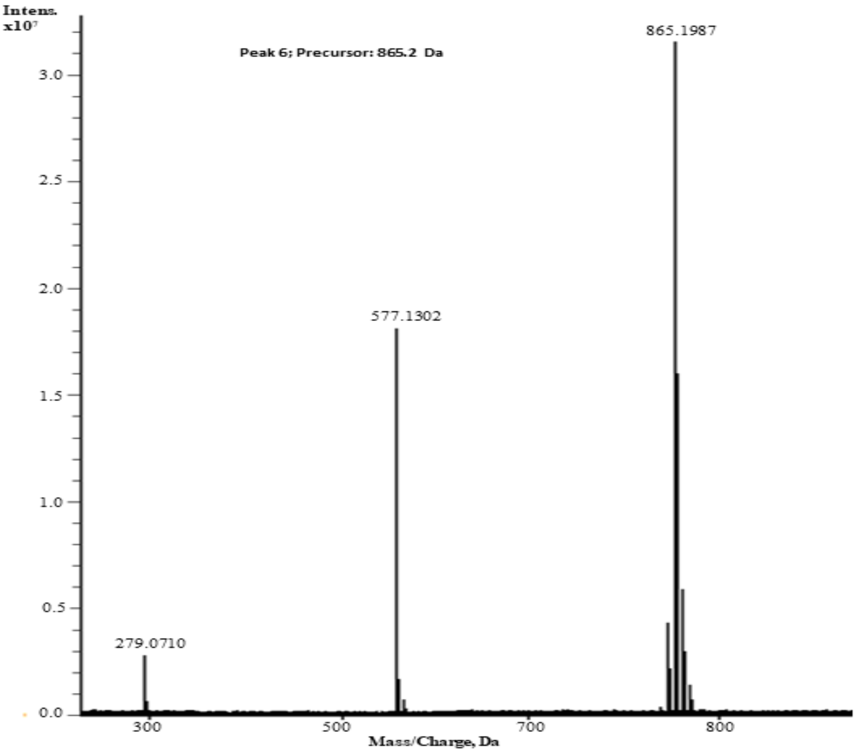

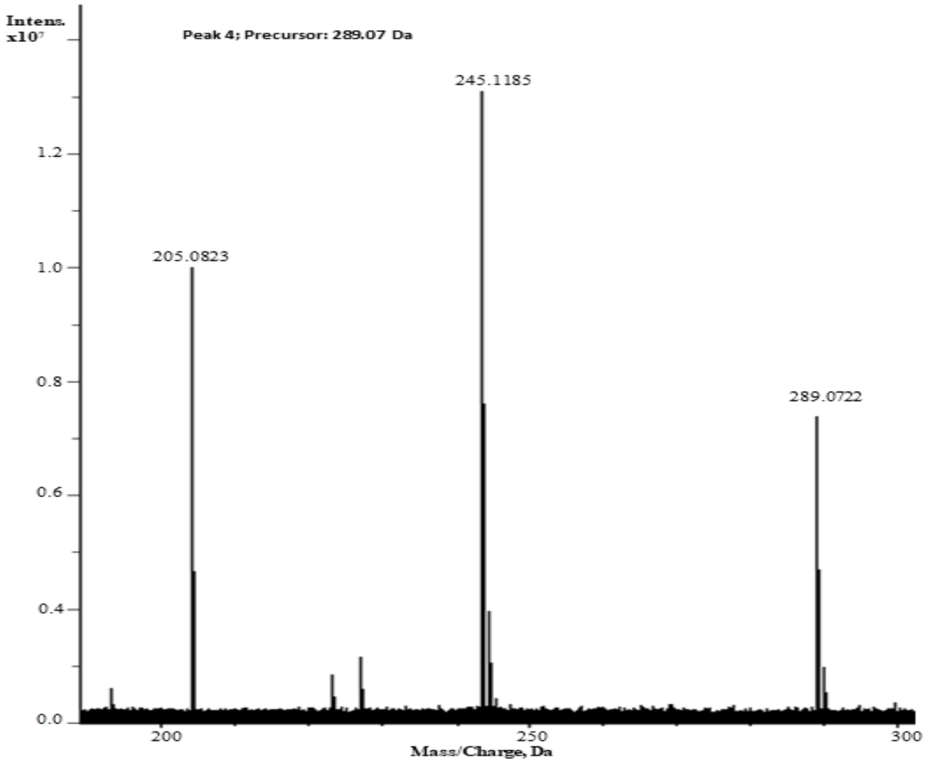

Figure S2 (continued)

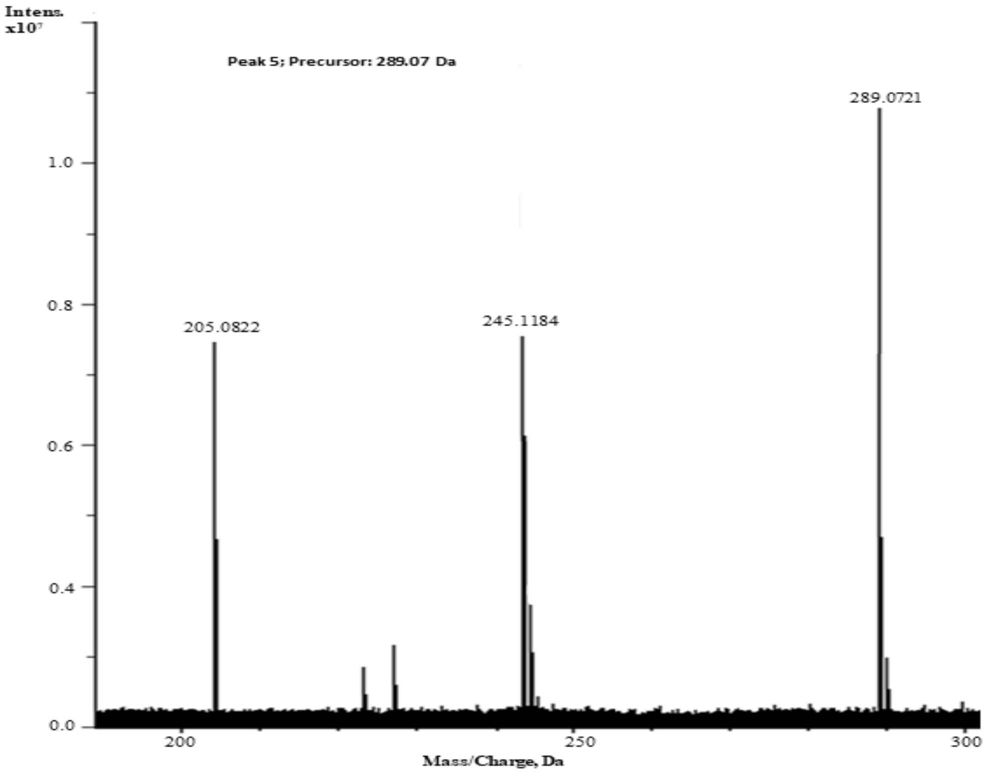

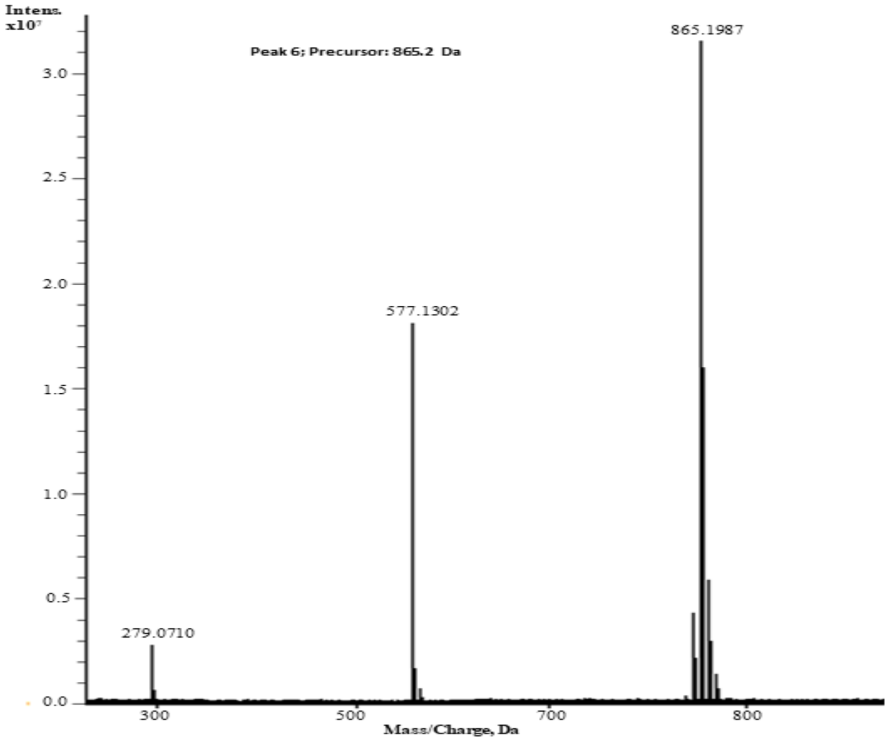

Figure S2 (continued)

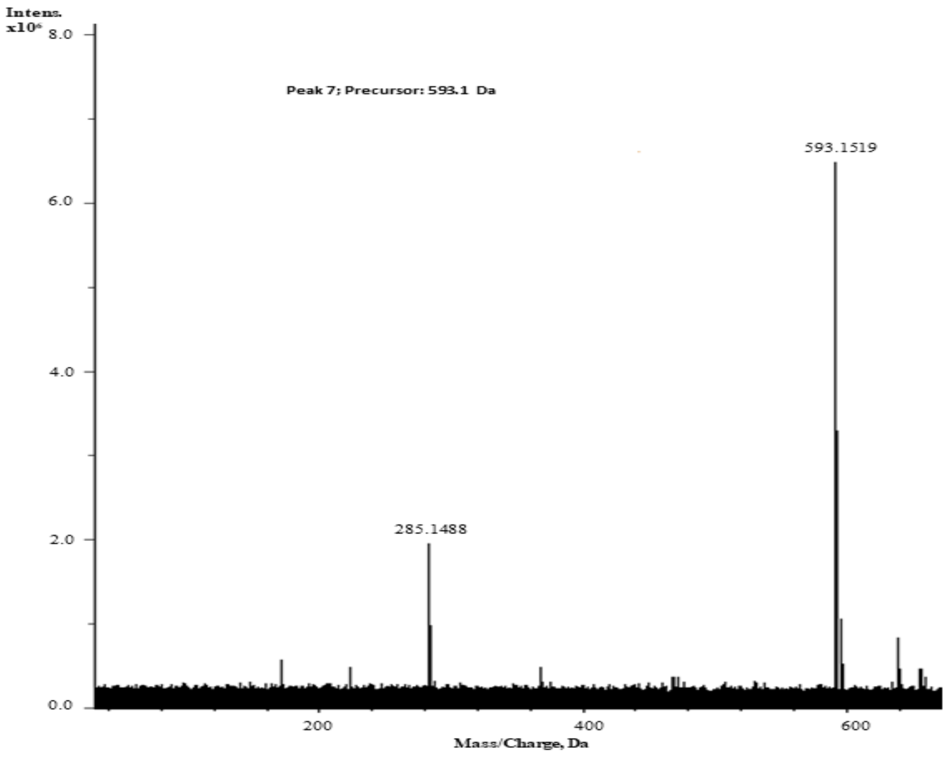

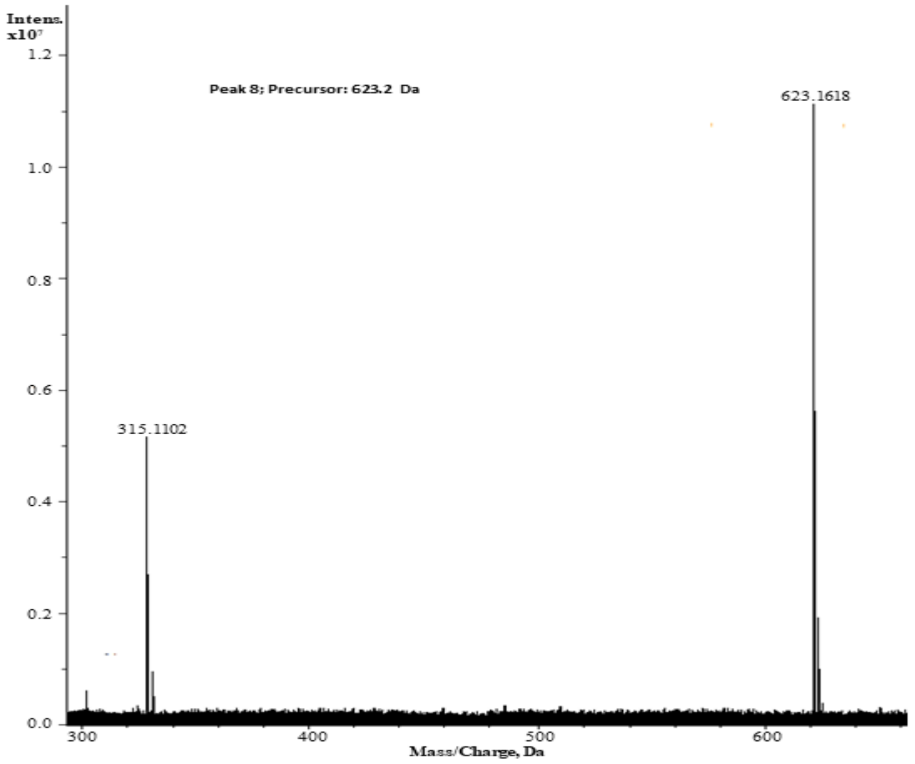

Figure S2 (continued)

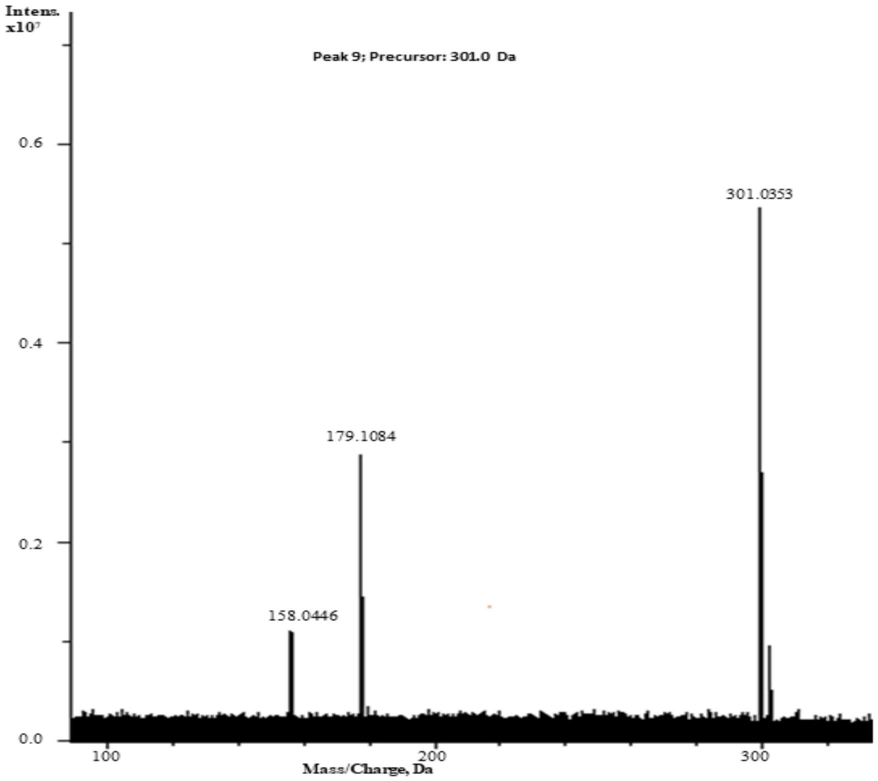

Figure S2. MS/MS spectra of polyphenol compounds in DSA. The peaks number is accordance with those in Figure S1

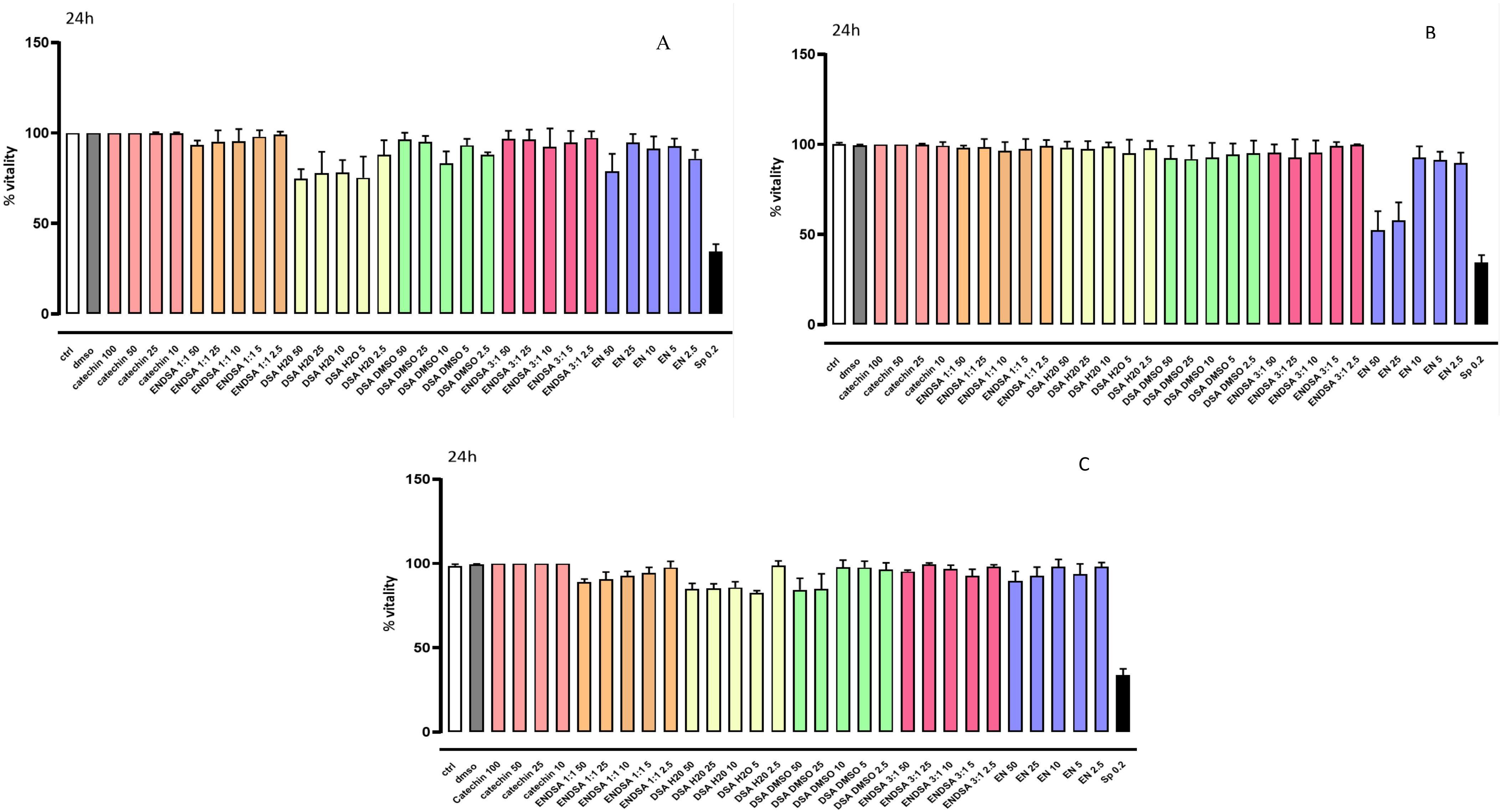

Figure S3. Cells vitality on A375 (A), A549 (B) and HaCaT (C) cells was calculated as: % vitality = [100 × (OD treated/OD control)]. Results were analysed by non-parametric Mann–Whitney U t

Table S1: %RH ± SD (n=3) of processed (40°C±2°C) and unprocessed samples

	**%RH of processed samples±SD**	**%RH of unprocessed samples±SD**
R1	8,05±0.10	58,63±1.25
R2	8,13±0.15	58,87±1.50
R3	8,09±0.25	57,96±1.25
Mean	**8,09±0.25**	**58,49±1.50**

R1: sample 1; R2: sample 2; R3: sample 3; %RH: percentage of Relative humidity SD: standard deviation
